# Subthreshold Operation of Organic Electrochemical Transistors for Biosignal Amplification

**DOI:** 10.1002/advs.201800453

**Published:** 2018-07-04

**Authors:** Vishak Venkatraman, Jacob T. Friedlein, Alexander Giovannitti, Iuliana P. Maria, Iain McCulloch, Robert R. McLeod, Jonathan Rivnay

**Affiliations:** ^1^ Department of Biomedical Engineering Northwestern University 2145 Sheridan Road Evanston IL 60208 USA; ^2^ Simpson Querrey Institute for BioNanotechnology Northwestern University Chicago IL 60611 USA; ^3^ Department of Electrical Computer, and Energy Engineering University of Colorado Boulder CO 80309‐0425 USA; ^4^ Department of Chemistry Imperial College London London SW7 2AZ UK; ^5^ Physical Sciences and Engineering Division KAUST Solar Center (KSC) King Abdullah University of Science and Technology (KAUST) Thuwal 23955‐6900 Saudi Arabia

**Keywords:** electroencephalography (EEG), organic electrochemical transistors, subthreshold, voltage amplifiers

## Abstract

With a host of new materials being investigated as active layers in organic electrochemical transistors (OECTs), several advantageous characteristics can be utilized to improve transduction and circuit level performance for biosensing applications. Here, the subthreshold region of operation of one recently reported high performing OECT material, poly(2‐(3,3′‐bis(2‐(2‐(2‐methoxyethoxy)ethoxy)ethoxy)‐[2,2′‐bithiophen]‐5‐yl)thieno[3,2‐*b*]thiophene), p(g2T‐TT) is investigated. The material's high subthreshold slope (SS) is exploited for high voltage gain and low power consumption. An ≈5× improvement in voltage gain (*A*_V_) for devices engineered for equal output current and 370× lower power consumption in the subthreshold region, in comparison to operation in the higher transconductance (*g*_m_), superthreshold region usually reported in the literature, are reported. Electrophysiological sensing is demonstrated using the subthreshold regime of p(g2T‐TT) devices and it is suggested that operation in this regime enables low power, enhanced sensing for a broad range of bioelectronic applications. Finally, the accessibility of the subthreshold regime of p(g2T‐TT) is evaluated in comparison with the prototypical poly(3,4‐ethylenedioxythiophene):poly(styrenesulfonate) (PEDOT:PSS), and the role of material design in achieving favorable properties for subthreshold operation is discussed.

## 1 Introduction

Electrophysiological monitoring has been a key tool for health care monitoring and clinical treatment for conditions^1^, ^2^ like epilepsy and Parkinson's disease as well as for creating brain–computer interfaces.^3^ Such activity is monitored by recording minute voltages associated with neural activity.^4^ Passive electrodes are the most common type of transducer for measuring such signals, which are then amplified and processed externally.^5^, ^6^ Further enhancement, however, could be achieved with an active transducer right at the recording site; organic electrochemical transistors (OECTs) are desirable candidates for such active recording elements.^7^, ^8^

OECTs are three terminal electrolyte‐gated devices with output and transfer characteristics similar to that of many other organic and inorganic semiconductor transistors, however, with a fundamentally different operating principle.^9^, ^10^ They consist of a (semi)conducting polymer channel in between a source and drain electrode, which is in direct contact (i.e., no dielectric layer) with surrounding electrolyte. The channel material's conductivity (doping level) changes due to ionic drift and associated injection of holes on application of a suitable gate potential. Local potential fluctuations in the biological tissue modulate the effective gate voltage of the OECTs and hence the doping state in the bulk of the channel. This modulation in the effective “gate” potential is typically transduced/measured by recording the transistor's drain current. Facilitated by the fact that these devices operate under low voltage, this principle has been exploited in numerous biological sensing applications.^11^ OECTs can be fabricated by conventional microfabrication techniques or other low temperature processing techniques like inkjet and screen printing;^11^, ^12^, ^13^, ^14^ many of these techniques are also very large scale integrated (VLSI) circuit compatible for manufacturing and designing dense arrays for high resolution sensing applications. Furthermore, OECTs can be manufactured on flexible substrates and of materials that are biocompatible and ultraconformable, which are desirable in in vivo sensing.^15^, ^16^ These unique characteristics of OECTs make them promising candidates for most bioelectronic sensor and stimulation applications.

OECTs are usually biased in a common source configuration, where the source terminal is grounded, the drain is connected to a source measure unit (SMU), and the gate electrode (often biased with a voltage source) is immersed in an electrolyte solution.^7^ Upon application of a bias at the drain (*V*_D_), a drain current (*I*_D_) flows through the device, the magnitude of which dictates the operating point of the transistor. This current is modulated when a signal voltage is applied at the gate of the DC‐biased transistor which results in ion‐induced (de)doping. The efficiency of this modulation can be indicated by the device transconductance, *g*_m_ =  ∂*I*_D_/∂*V*_G_. This parameter is determined from the slope of the transfer characteristics (*I*_D_ vs *V*_G_) or by applying a small sinusoidal signal voltage on the gate and measuring the resulting sinusoidal amplitude in the current (small signal transconductance). Unlike a traditional inorganic metal oxide semiconductor field‐effect transistor (MOSFET), OECTs have been shown to exhibit a nonmonotonic *g*_m_ over the applied gate voltage. Typically, a “peak *g*_m_” region can be identified, and the device is usually operated in this regime.^17^ Several works have studied this region of operation, focusing on optimized device dimensions and materials to enhance performance.^18^, ^19^ Some of the materials developed via these efforts^20^, ^21^ have shown that a subthreshold swing of 60 mV dec^−1^ is possible without the need for complex/careful device design and without defect/interface engineering. So far, however, there have been no efforts to operate OECT biosensors in the subthreshold regime.

Subthreshold region of operation in MOSFETs has been of considerable interest. One of the main reasons is that in this regime, the transconductance efficiency (*g*_m_/*I*_D_), i.e, *g*_m_ obtained per unit current is very high due to the exponential current–voltage (*I*_D_ − *V*_G_) characteristics.^22^, ^23^ This leads to increased power efficiency and hence has been critical in low power electronics applications.^24^, ^25^, ^26^ The subthreshold regime often occurs at low current (*I*_D_), however, geometrical scaling of transistors allows one to engineer a device with higher currents to achieve a desired gain, while at the same time being power efficient. This is important for implantable biosensors where power consumption is of vital importance.^27^, ^28^ The power dissipation in the form of heat can also affect the tissue surrounding the device.^29^ Due to these factors, MOSFET‐based amplifiers in medical implants have used the subthreshold region of operation.^30^, ^31^

Herein, we demonstrate the use of p(g2T‐TT)^21^ OECTs in the subthreshold region as voltage amplifiers. We compare this region of operation with the traditional peak *g*_m_ region in terms of improvements in voltage gain, current gain efficiency, and power consumption. We also compare its performance with that of the prototypical conducting polymer material poly(3,4‐ethylenedioxythiophene) complexed with poly(styrenesulfonate), PEDOT:PSS, and discuss material characteristics that enable efficient subthreshold operation. We apply a device modeling approach of material parameters to investigate the practical accessibility of the subthreshold regime in OECTs. Finally, we show that subthreshold operation can be used to measure low amplitude electroencephalography (EEG) signals, highlighting the potential future in low power biosensing capabilities.

## 2 Results and Discussion

**Figure 1:**
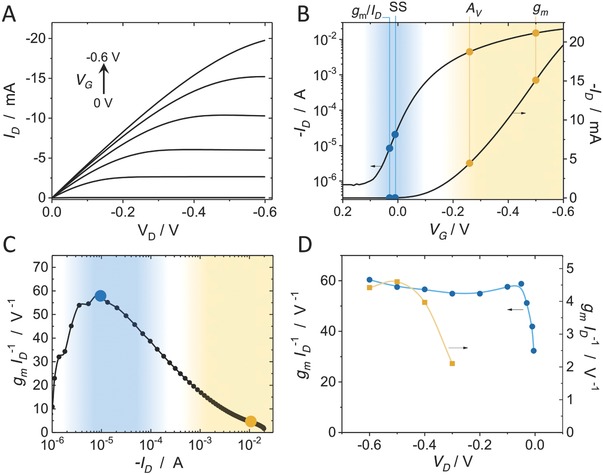
Characteristics and operating regions of a 100 × 10 µm^2^ (*W* × *L*), and 200 nm thick p(g2T‐TT) device: A) output (*I*_D_–*V*_D_) characteristics; B) transfer (*I*_D_–*V*_G_) characteristics at *V*_D_ = −0.6 V in the linear scale (right axis), and log scale (left axis). The operating points of peak transconductance (*g*_m_), peak voltage gain (*A*_V_), peak subthreshold slope (SS), and peak transconductance efficiency (*g*_m_/*I*_D_) are noted. C) Transconductance efficiency, *g*_m_/*I*_D_, versus *I*_D_ (the orange and blue points denote the operating conditions for (D). D) Transconductance efficiency versus drain voltage (*V*_D_); the *I*_D_ associated with peak *g*_m_ efficiency at *V*_D_ = −0.6 V (≈10 µA), blue circles, and at the *I*_D_ of peak *g*_m_ (≈10 mA), orange squares. The regions and operating points associated with the subthreshold region of operation are shown in blue, and those of the superthreshold region in orange.

OECTs based on p(g2T‐TT) as the active channel material are hole transporting (p‐type) enhancement mode devices. In such devices, the application of a negative gate voltage (*V*_G_) enables oxidation of the semiconducting polymer channel in order to turn on the device. **Figure** **1**A shows typical output (*I*_D_–*V*_D_) characteristics of a *W* = 100 µm, *L* = 10 µm device with 200 nm polymer layer thickness at various gate voltages, and Figure 1B shows the transfer characteristics in log and linear scale. Slope in the linear scale represents the transconductance, *g*_m_, and exhibits a peak value of *g*_m_ = 50 mS at *V*_G_ = −0.48 V (the location is shown in Figure 1B). The subthreshold region can be visualized in a log scale plot with the location of maximum slope (60 mV dec^−1^, at *V*_G_ ≈ 0 V). The device operates within the subthreshold regime from ≈100 to 1 µA. Also shown in Figure 1B is the location of peak voltage gain, which depends on how deep into saturation the device is operated (i.e., the output impedance), as discussed below. For p(g2T‐TT), it is evident that the subthreshold region occurs at low gate voltage offset, due to the accumulation mode of operation, which is a desirable trait for implanted biosensors.

In the subthreshold region, the transconductance efficiency, *g*_m_/*I*_D_, is highest and is a well‐established property in MOSFETs.^32^ Our devices exhibit similar characteristics (Figure 1C) with high transconductance efficiency (peaking at 60 V^−1^ at *I*_D_ = 10 µA or *V*_G_ = 50 mV) in the subthreshold region. This parameter directly dictates the power consumption by the device. Figure 1D shows that the region of high transconductance efficiency is approximately constant over a broader range of drain voltages. This can also be inferred from subthreshold transfer characteristics obtained at various drain voltages (Figure S1, Supporting Information). In contrast, when operating a device at currents associated with the high *g*_m_ region, both transconductance and transconductance efficiency deviate as the device leaves the saturation regime. Additionally, it is well established that the peak transconductance magnitude and its associated gate voltage show a strong *V*_D_ dependence.^17^

In the subthreshold region, owing to the exponential nature of the *I*–*V* characteristics, the *g*_m_ behaves as follows^23^

(1) $$g_{m}\;=\;\frac{q}{nkT}\;\times \;I_{D}$$

The *q*/*nkT* term can be obtained from the slope of the transfer curve (at the operating point) in the natural log scale. The calculated value from the above equation and is ≈1.5 mS at peak subthreshold slope (SS) (*V*_G_ = 0 V), which is in agreement with the first derivative of the transfer curve *g*_m_  =  ∂*I*_D_/∂*V*_G_ at the same operating point (1.5 mS). It should be stressed that the above equation is only relevant for the subthreshold region of operation. In such a region, the *g*_m_, and hence gain, varies proportional to *I*_D_, in contrast to $\sqrt{I_{D}}$ in superthreshold region.^23^

**Figure 2:**
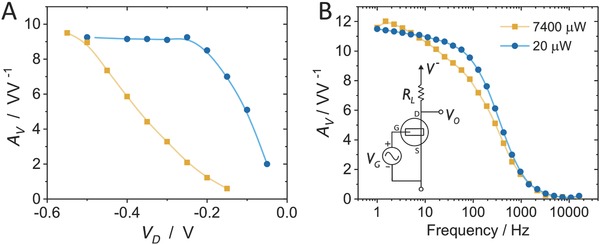
Performance comparison of subthreshold (blue circles) and superthreshold (orange squares) regimes. A) Voltage gain as a function of drain voltage while operating at subthreshold or peak voltage gain (at *V*_D_ = −0.6 V), by varying the load resistor (*R*_L_). The subthreshold device maintains the same gain at much lower *V*_D_. B) Voltage gain bandwidth shows similar results for both regions, however subthreshold operation consumes ≈370× less power.

Proper comparison of the performance of the OECT in the subthreshold and superthreshold regimes depends largely on the desired characteristics when integrated into a circuit, including voltage gain and power consumption. To compare power consumption, we set the voltage gain (*A*_V_) at the two regimes to be the same by varying the magnitude of a load resistor, *R*_L_ (**Figure** **2**B, inset). This is because the inherent *g*_m_ is low at the subthreshold region due to low *I*_D_ and hence requires higher *R*_L_ for the same *A*_V_ (*A*_V_ = *g*_m_ × *R*_L_). At subthreshold, the device is operated at *V*_G_ = 0 V and at superthreshold, *V*_G_ = −0.25 V. At superthreshold, the device is operated at *I*_D_ ≈ −3.5 mA rather than −15 mA (*V*_G_ = −0.48 V) which corresponds to the peak transconductance, because the output impedance *R*_o_ must be taken into account (see Figure S3 in the Supporting Information) and defines the intrinsic maximum achievable gain of *g*_m_ × *R*_o_. As such, the peak intrinsic gain region is found to occur at *V*_G_ = −0.25 V. Load resistors were chosen to fix the extrinsic voltage gain at ≈10 (arbitrarily chosen and at 10 Hz), with *R*_L_ = 10 kΩ at subthreshold, and 0.43 kΩ at superthreshold. Under these load and biasing conditions, *A*_V_ for different *V*_D_ and bandwidth are measured (∆*V*_o_/∆*V*_G_), Figure 2A,B, respectively.

As designed, the voltage gain at *V*_D_ = −0.6 V is equal, however, the gain at subthreshold is maintained over a wider range of *V*_D_, Figure 2A, compared to operation at superthreshold – in agreement with Figure 1D. For the power analysis, the *V*_D_ is selected to be as low as possible, but maintaining the same *A*_V_ (0.25 and 0.6 V for sub‐ and superthreshold, respectively). The bandwidth plots (Figure 2B) reveal minimal difference between the two cases for frequency dependence which is of important consideration in electrophysiology.^33^ The power consumed was calculated by the *V* ^−^ ×*I*_D_ product,^34^ which is the average DC power consumed by the circuit for amplification. Subthreshold operation consumed ≈370× less power than the high *A*_V_ region for the same gain output (20 µW vs 7.4 mW). This power consumption is effectively dissipated as heat and hence lower power would cause lower heat exposure to the tissue, supporting the notion that the subthreshold operating OECT could offer a safer alternative than peak *g*_m_.

The drain current predominantly dictates the operating point of a discrete transistor‐based amplifier system and hence for fair comparison of the gain, the same biasing condition should be applied. The challenge here is that both regions show significantly different current levels. Hence, we engineer two different devices by changing *W*/*L* and thickness, *d*, in order to access the two regimes at approximately the same current (Figure S2 and “Supporting Method” Section, Supporting Information). This approach shows that operating at the same current (100 µA), a thinner (≈50 nm) and smaller *W*/*L* device (*W*/*L* = 1) is in the superthreshold regime with *g*_m_ = 0.6 mS, while the thicker (≈200 nm) and *W*/*L* = 10 device operates in subthreshold at *g*_m_ = 3.5 mS. This time, keeping the same load resistor, we can confirm that voltage gain is 5× larger for the subthreshold device, and is again attributed to the *g*_m_ efficiency (*g*_m_/*I*_D_) discussed above, Figure S2C (Supporting Information).

**Figure 3:**
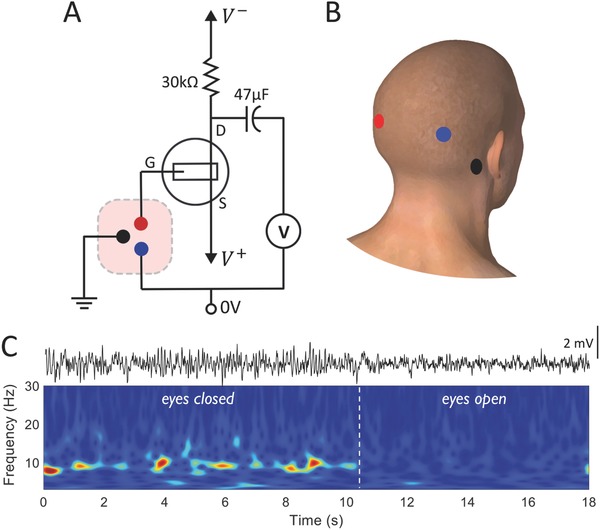
EEG sensing using a subthreshold OECT voltage amplifier. A) Voltage amplifier circuit wiring diagram for operating the OECT in the subthreshold regime for EEG measurement. B) Electrode placement on the scalp of a human subject color coded corresponding to circuit connections in (A). C) EEG voltage output signal measured in the subthreshold region showing alpha rhythm response (top), and the corresponding time–frequency plot (bottom) showing changes in signal content depending on the eyes being closed and opened. Typical 10 Hz activity, alpha rhythms, are observed in the eyes closed condition.

While the above gain and power analysis is helpful to quantify the operation of OECTs, an illustrative example of subthreshold electrophysiological sensing, for example EEG, further motivates the need to target and design devices and circuits to take advantage of this less explored operation regime in OECTs. Similar to the voltage gain analysis, we chose an operating current of 100 µA to make sure that there was enough gain to record the physiological oscillations. This requires a gate bias and hence we created a voltage amplifier, as shown in **Figure** **3**A, by biasing the source terminal (*V* ^+^) instead, to enable direct coupling of the gate electrode with a subject's head (the EEG source) as in Figure 3A. We added an output coupling capacitor to eliminate the DC signal and hence have maximum AC measuring sensitivity for transducing the low amplitudes of EEG (10–100 µV). The source voltage (≈50 mV) was adjusted to set the operating current close to 100 µA, subthreshold region, and deeper into saturation (with voltage between source and drain, *V*_D_ > 0.4 V), using a load resistor of *R*_L_ = 30 kΩ; our voltage gain was 30. Adhesive medical electrodes from the scalp (Figure 3B) were connected externally to the OECT and to ground, as noted in Figure 3A (see the Experimental Section for further details). Figure 3C shows a voltage trace measured from this circuit, as well as the corresponding time–frequency plot. The recorded segment shows an interval whereby the subject's eyes were closed, where alpha rhythm (≈10 Hz) activity, indicative of wakeful relaxation, is readily observed. Upon opening eyes, the observed alpha rhythms immediately subside. The power spectral density of the signal content in the two conditions is shown in Figure S4 (Supporting Information). The alpha waves had an amplitude of ≈1–2 mV peak‐to‐peak, obtained due to the amplification by the OECT circuitry. EEG signals were also measured by direct voltage recording of biopotentials using the same medical electrodes (Figure S5, Supporting Information), showing the 10 Hz alpha rhythm activity when eyes are closed, this time recorded by conventional means without the OECT circuit. In contrast to the amplified signals, those from the direct voltage recordings were ≈20–50 µV peak‐to‐peak. The electrophysiological nature of the recorded signals is further validated by measuring electrocardiography (ECG) using the subthreshold OECT amplifier circuit, Figure S6 (Supporting Information), which produces the expected ECG waveforms. In this work, we do not perform a first‐hand comparison of the sensing in the two regimes, but instead, show that operation in subthreshold is possible, and worthy of further exploration, given the device and circuit analysis described above.

The results and discussion above are enabled by the steep subthreshold characteristics of the p(g2T‐TT) device. An important consideration when operating in subthreshold is the stability of the threshold conditions in response to operation. We find minimal effects of such stressing on the threshold stability: continuous pulsing of an OECT for over 30 min results in small changes in drain current at deep subthreshold (Figure S7, Supporting Information).

**Figure 4:**
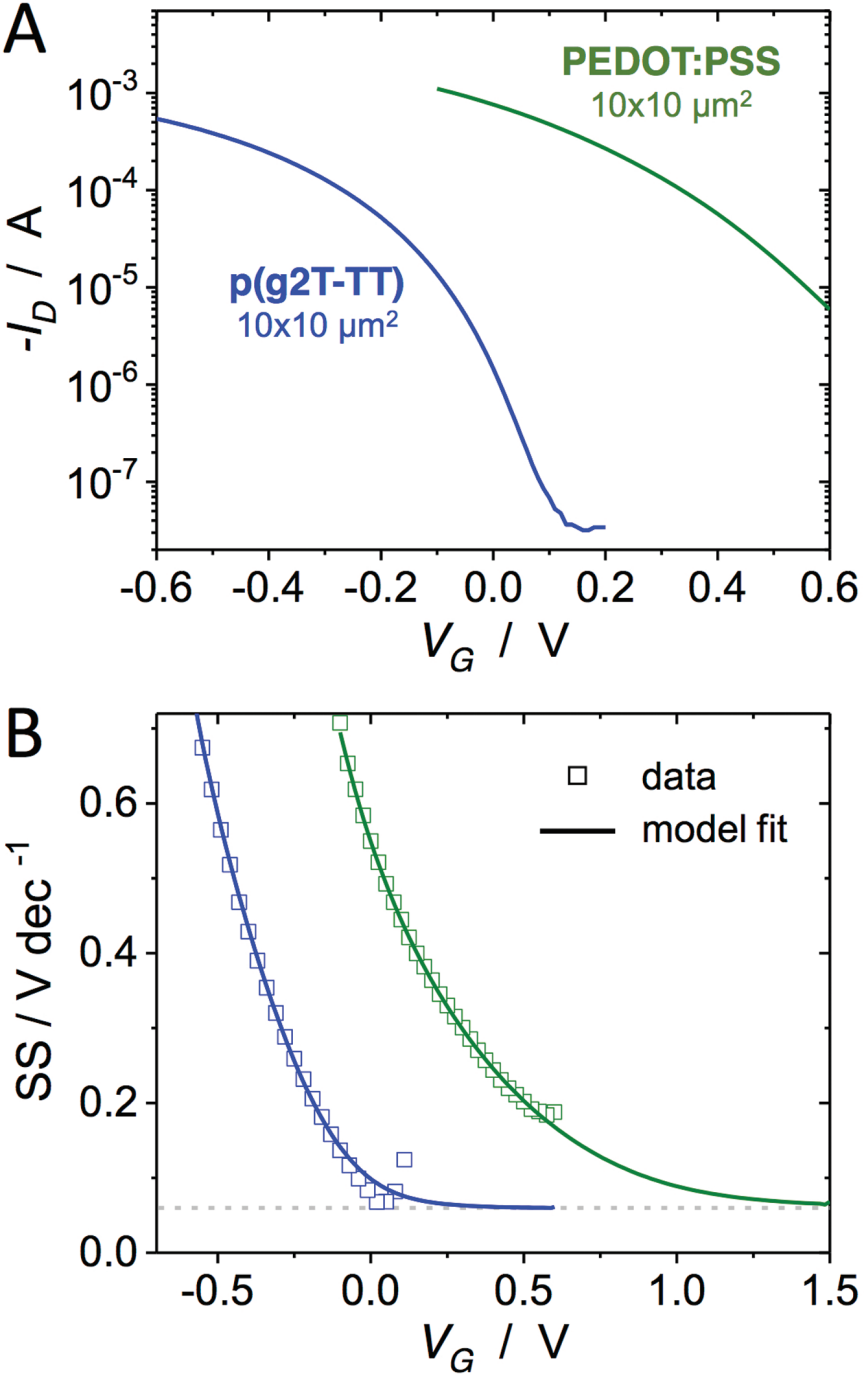
Material comparison and subthreshold accessibility. A) Transfer characteristics of p(g2T‐TT) (10 × 10 µm^2^, 50 nm thick, blue) with PEDOT:PSS (10 × 10 µm^2^, 100 nm thick, green) at *V*_D_ = −0.6 V. B) The same data plotted as subthreshold swing (SS), with fit result from the disorder model of ref. 35. The gray dotted line denotes 60 mV dec^−1^.

The accessibility of the subthreshold regime, however, is not universal, especially when operating in aqueous conditions where biasing magnitudes are restricted due to hydrolysis. It is worthwhile to therefore investigate the role of material design in subthreshold operation. We compared the characteristics of the p(g2T‐TT) devices discussed herein to those of PEDOT:PSS. Typical of such depletion materials, PEDOT:PSS requires a positive gate voltage to fully dedope the material and decrease the drain current into the subthreshold region. This means higher operating voltages would be required to operate in this regime. **Figure** **4** shows the transfer characteristics as well as the voltage swing (in mV dec^−1^) of both materials. From a material perspective, it is clear that material design enables engineering of the gate bias of subthreshold operation.

In conventional MOSFETs, the subthreshold characteristics are dictated by a SS with a lower bound of 60 mV dec^−1^. As shown in Figure 4B, p(g2T‐TT)‐based OECTs can easily reach this limit. The model of Friedlein et al.^35^, ^36^ suggests that the lower bound of the SS in OECTs is governed by the same physics as in crystalline FETs, and that 60 mV dec^−1^ is theoretically achievable not only for p(g2T‐TT), but also for other OECT materials. Figure 4B shows that the fits to the transfer curves predict an asymptote to 60 mV dec^−1^ in both materials. In the case of PEDOT:PSS, the fits show that while a 60 mV dec^−1^ SS can be attained, it only approaches this value at *V*_G_ near 1.5 V, and thus the subthreshold is less readily accessible. In addition to material design to control native doping state, recent work has shown that chemical control of the electrochemical potential at the gate electrode can be used to effectively shift the transfer characteristics of a given material.^37^

The relative ease with which the studied OECTs can reach 60 mV dec^−1^ may be due to a balance between the efficient trap filling associated with ion doping and maintaining a low enough doping concentration in subthreshold to avoid potential effects due to large‐scale mass injection and the disorder therein. Further understanding of material contributions in the subthreshold may be enabled by advancements in device modeling taking into account disorder: while such parameters can be extracted from the model employed in Figure 4B, their absolute values and trends therein should be further studied in depth. Finally, it is desirable for the subthreshold regime to be maintained over a broad gate voltage range, in the case of the OECTs, this appears to be a challenge due to the relatively high *I*_D_ off currents, which are found to be on par with^21^ or within an order of magnitude of the gate current, *I*_G_ (Figure S8, Supporting Information). This suggests that a better understanding of the deep subthreshold, OECT off current, and its relation to gate current may allow for an extended subthreshold range with broader accessibility.

## 3 Conclusion

A detailed analysis of the subthreshold region of operation of p(g2T‐TT) OECTs as voltage amplifiers has been reported. Emphasis was placed on studying the exponential voltage gain and low power consumption in these regions. The high transconductance efficiency was identified as a critical characteristic for the OECTs studied, and it explains a variety of the circuit‐based comparisons described herein. A 5× improvement in voltage gain was established by operating the device in the subthreshold region at the same DC bias conditions (same *I*_D_). Finally, a power analysis was performed for circuits with the same voltage gain, and revealed a 370× reduction in power consumption when the device is operated at the subthreshold region. SS operation for electrophysiological applications was demonstrated through EEG measurements using p(g2T‐TT) OECT‐based voltage amplifier. Device modeling suggests that SS = 60 mV dec^−1^ is possible for different OECT materials, but that device and materials engineering is required to access the deep and broad subthreshold regime within safe operation limits. In addition, operation in the lower current subthreshold regime is expected to minimize the effects of resistive heating, Faradaic reactions, and potential contact effects, and will put less stress on the active material, potentially leading to more stable operation with less degradation.^21^ These findings represent device and material design/selection criteria for subthreshold sensing with OECTs.

The relative ease with which a device with stable subthreshold at 60 mV dec^−1^ is fabricated with the present materials is of particular note. The active materials, spun cast from solution, provide robust devices that can be integrated into large‐scale, high density arrays and utilized in biosensing applications while simultaneously taking advantage of lower power consumption and high voltage gains. While the demonstration and analysis in this work motivate further investigation, system level design of OECT‐based sensing circuits^37^ must be well thought out when considering subthreshold implementation. For example, to achieve the same *A*_V_, due to low *g*_m_ of the subthreshold region, a larger *R*_L_ is required, which can pose difficulties for circuit design and can contribute to higher thermal noise. Future work should address these aspects, and target subthreshold circuits with OECTs for creating on‐site amplification in implantable devices. Further, given the exponential *I*–*V* nature at the subthreshold region, translinear circuits can be realized to achieve other analog processing functions like multiplication and filtering, which also have desirable applications for signal processing.^38^

## 4 Experimental Section

*Device Fabrication*: The device was fabricated as previously reported.^39^ Briefly, glass slides (1 × 3 in.) were thoroughly cleaned by mechanically rubbing in soap solution, rinsing with deionized (DI) water, sonicating in baths containing acetone and isopropanol and drying with nitrogen. The source, drain, and interconnects of gold were patterned using a lift‐off process. Positive photoresist S1813 was spin‐coat‐deposited, exposed using MJB4 mask aligner, and developed using the AZ400K developing solution followed by e‐beam deposition of Au of 100 nm with Cr adhesion layer (2 nm). Liftoff was done using microposit photoresist strip 1165. Two parylene layers were then deposited sequentially using an SCS Labcoater II. The first layer acts as insulation for the interconnects, while the second layer is a sacrificial layer for patterning the active polymer; the layers are separated by a layer of Micro‐90, to allow for facile peel off of the sacrificial parylene. A RIE etching system was used to define the channels and expose contact by etching the parylene (O_2_, CHF_3_), with a thick photoresist (AZP4620) as the mask patterned using a second photolithography step.

Polymer solutions containing the active materials were prepared as follows for spin‐coating process. p(g2T‐TT) was dissolved in chloroform at concentration of 5 mg mL^−1^ at 35 °C. The solution was then spin‐coated at 1000 rpm, 30 s resulting in a thickness of about 50 nm film. For the thicker film, a slow speed of 200 rpm was chosen, resulting in a thickness of about 200 nm. The PEDOT:PSS formulation was 94 wt% PH‐1000, 5 wt% ethylene glycol, 1 wt% (3‐glycidyloxypropyl)trimethoxysilane (3‐GOPS), 0.1 wt% dodecyl benzene sulfonic acid (DBSA). The spin recipe was 3000 rpm, 1500 rpm s^−1^, 30 s, and was annealed at ≈125 C, 1 h. The resulting PEDOT:PSS film was 100 nm. The sacrificial parylene was then peeled, resulting in the patterned active layers in the channels. The finished devices were rinsed with DI water to remove the soap, and contact pads were cleaned using clean‐room swabs with acetone or water. Thicknesses were measured using a Veeco Dektak‐8 profilometer.

*Device Characterization*: A small self‐contained volume, ≈100 µL, of electrolyte (100 × 10^−3^ m NaCl in DI water) was added on top of the device area and a Ag/AgCl pellet (Warner Instruments) was dipped into the solution to act as the gate electrode. National Instruments (NI) SMUs [NI PXIe‐4143] were used for sourcing and measuring the drain–source voltage and current, and gate current. The gate voltage was applied using NI's data acquisition (DAQ) card [NI PXIe‐6363] and the output measured using a NI BNC‐2110. All measurements were automated using custom LabVIEW software. For gain calculations, curve fitting was performed using MATLAB software (Mathworks).

*EEG Experiments*: Three medical adhesive electrodes (3M Red Dot) were placed on the scalp of the human subject, two on the rear, behind the visual cortex and one on the mastoid process bone which was connected to the ground. The rear electrodes were connected to the OECT's gate and the low terminal (0 V) of the voltage source. The output voltage was measured using NI's PXIe‐4081 digital multimeter (DMM) automated using LabVIEW software, sampled at 1 kHz. Direct EEG voltage sensing (without OECT and circuit) was performed using the same 3M Red Dot electrodes wired directly to the NI PXIe‐4081 DMM. The data were then processed and filtered in MATLAB, applying bandpass (1‐100 Hz) and notch (55‐65 HZ) filters. Gabor wavelet analysis was then used to create the time‐frequency plots, and pwelch function was used to extract power spectral density. All participants provided informed consent for the experiment. Only medical‐grade electrodes (3M Red Dot, MFR#9640) were applied to the skin.

## Conflict of Interest

The authors declare no conflict of interest.

## Supporting information

Supplementary
