# Supplementary material for: Subthreshold Operation of Organic Electrochemical Transistors for Biosignal Amplification
Source: Adv Sci (Weinh). 2018 Jul 4;5(8):1800453. doi: 10.1002/advs.201800453 (PMC6097142; doi:10.1002/advs.201800453)
Supplement: Supplementary file 1 — Supplementary [file ADVS-5-1800453-s001.pdf]

Copyright WILEY-VCH Verlag GmbH & Co. KGaA, 69469 Weinheim, Germany, 2018.

ADVANCED SCIENCE
Open Access

Supporting Information

for *Adv. Sci.,* DOI: 10.1002/advs.201800453

Subthreshold Operation of Organic Electrochemical Transistors for Biosignal Amplification

*Vishak Venkatraman, Jacob T. Friedlein, Alexander Giovannitti, Iuliana P. Maria, Iain McCulloch, Robert R. McLeod, and Jonathan Rivnay**

Supporting Information

Subthreshold operation of organic electrochemical transistors for bio-signal amplification

*Vishak Venkatraman[1,2], Jacob T. Friedlein[3], Alexander Giovannitti[4], Iuliana P. Maria[4], Iain McCulloch[4,5], Robert R. McLeod,[3] and Jonathan Rivnay[1,2]**

Supporting Figures:

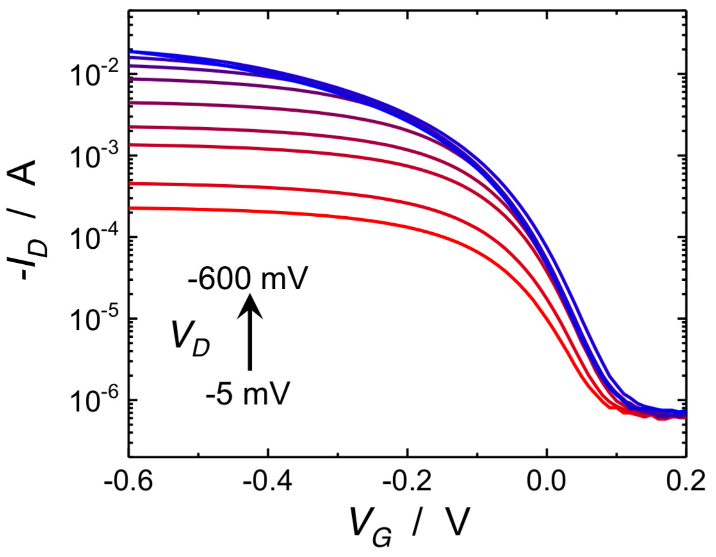

Figure S1. Transfer characteristics for 100 µm x 10 µm, 100 nm thick device at different drain voltages showing same subthreshold slope for a wide range of -V_D. (5, 10,30,50,100,200,300,400,500,600 mV)

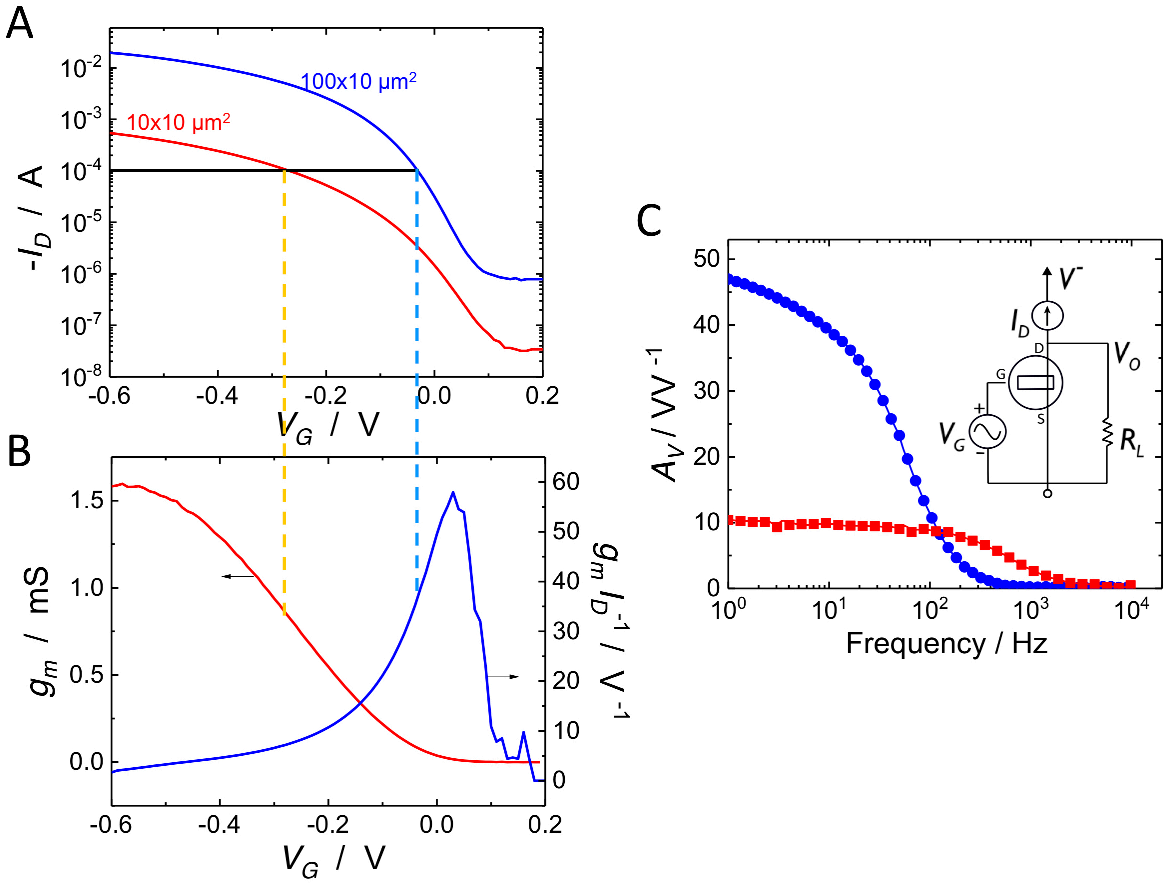

Figure S2. (A) Transfer curves of 100 µm x 10 µm (200 nm thick) OECT, blue, and 10 µm x 10 µm (50 nm thick) OECT, red. (B) Plots of transconductance, g_m, for the 10 µm x 10 µm device, left axis, and g_m efficiency for the 100 µm × 10 µm device. Solid black line denotes the constant operating current of 100 µA, and resulting voltage and g_m (orange dashed line), and g_m efficiency values (blue dashed line) for each device in their intended operation regimes (superthreshold for the 10 µm × 10 µm device, and subthreshold for the 100 µm × 10 µm device). (C) Voltage gain as a function of frequency for the two devices; inset: circuit diagram of the setup used, where voltage gain, $A_v = \Delta V_o / \Delta V_G$

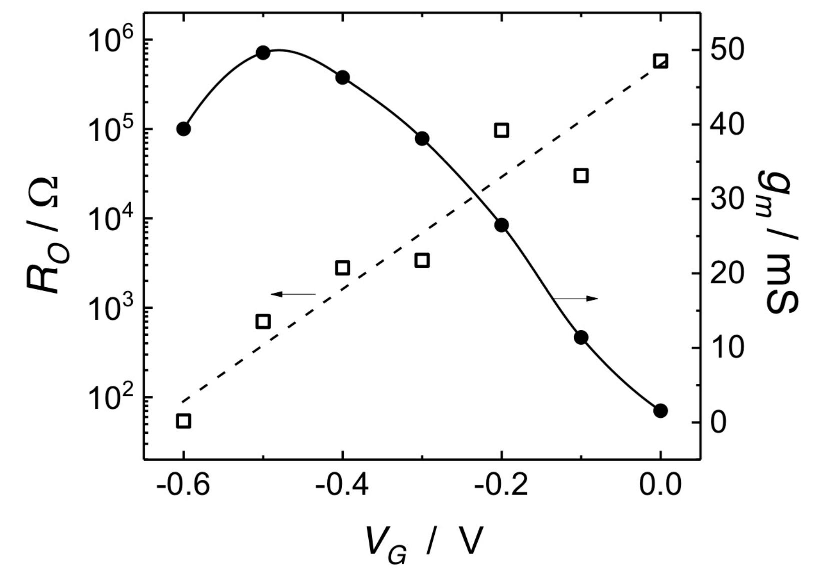

Figure S3. Output impedance, R_o, peak at different location than peak g_m, which affects the intrinsic voltage gain.

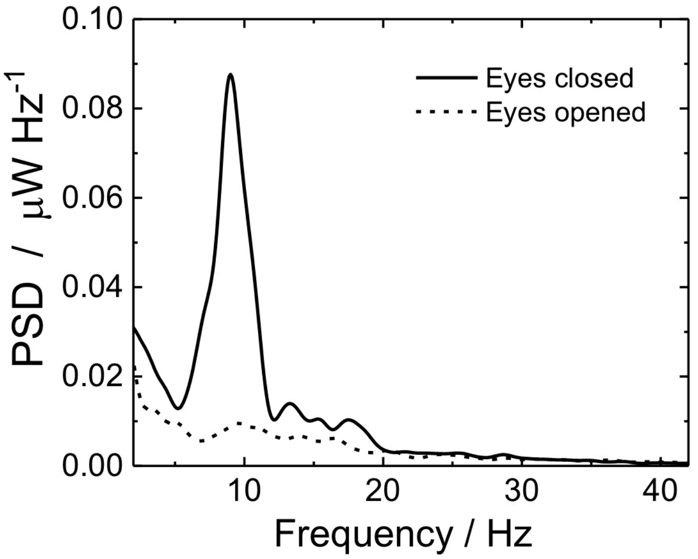

Figure S4. Power spectral density for EEG experiment in main text, for eye closed condition (observable 10 Hz alpha rhythms), and eyes opened.

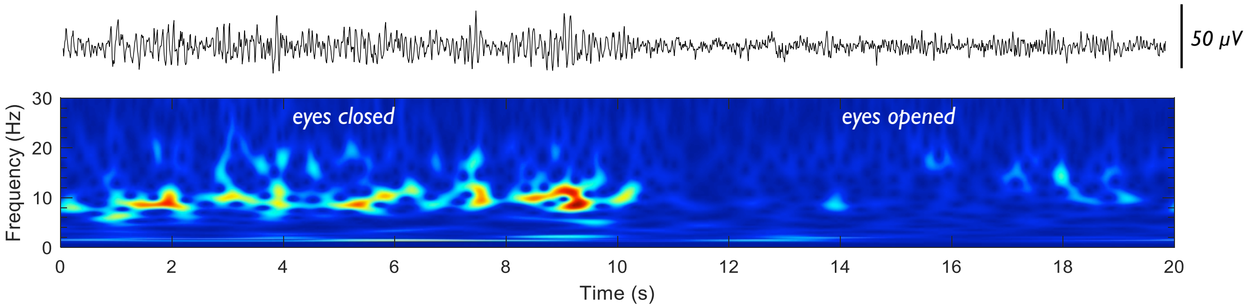

Figure S5. Time-frequency plot for EEG signals measured using 3M Red Dot adhesive medical electrodes recorded directly with the NI PXIe-4081 DMM, without the OECT or accompanying circuit. The periods of "eyes open" and "eyes closed" are noted for clarity. Note the voltage-time trace for the corresponding EEG signal of the DMM recording: the alpha wave amplitudes are ~20-50 μV peak-to-peak, as compared to the amplifier circuit, which measures the same signals at ~1-2 mV.

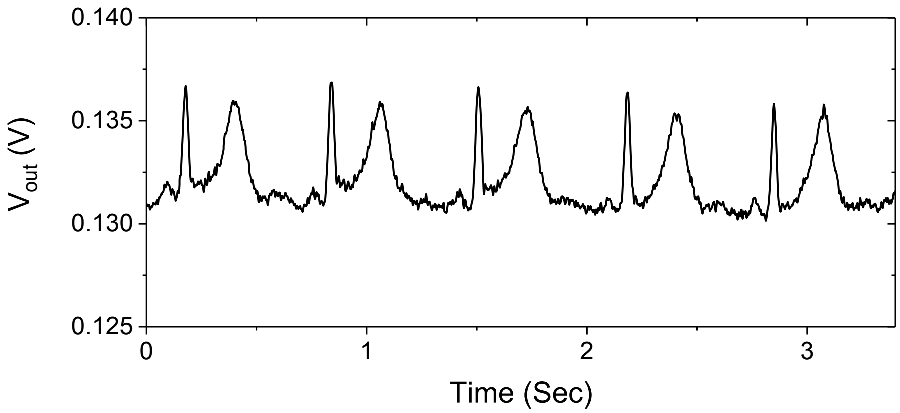

Figure S6. ECG signal recorded using OECT subthreshold amplifier circuit, using a feedback resistor of 19 kΩ. The 3M Red Dot medical adhesive electrodes are attached on either side of the chest, and connected to the gate and source terminals of the OECT.

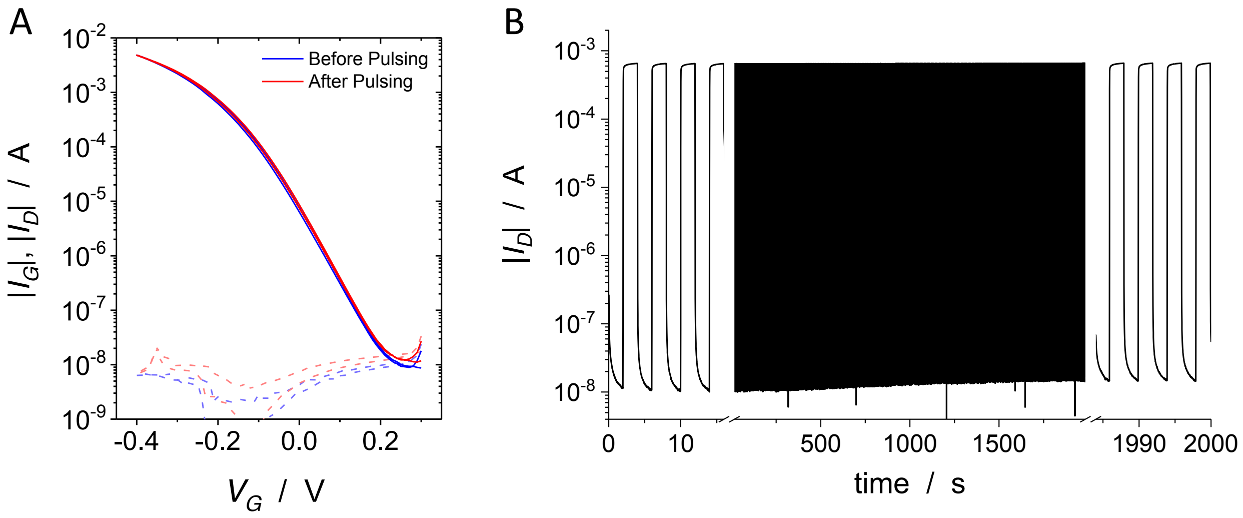

Figure S7. OECT stressing and threshold stability. A. Transfer characteristics (solid line: I_D, dashed line: I_G) before (blue) and after (red) continuous gate voltage pulsing for >30 minutes. V_D=-0.4 V. B. Drain current vs. time for the continuous pulsing experiment mentioned in (A). V_D=-0.4 V, with V_G pulsed between +0.3 V and -0.2 V, with a pulse duration of 2 s. This gate voltage range was selected to enable proper ranging of the current values without changing current range limits during acquisition with the NI PXIe SMUs. The observed shift in transfer characteristics after stressing is minimal, for example, I_D at V_G=0 V increases from 7.7 μA to 8.6 μA (a shift of ΔV_G=-3.9 mV) after 30 minutes of pulsed biasing.

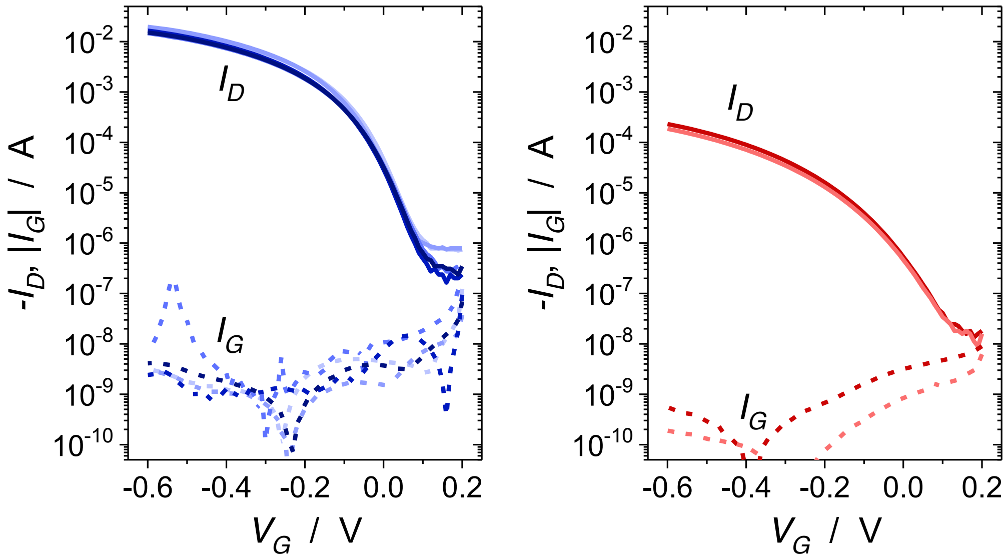

Figure S8. Transfer curves of 100 µm x 10 µm (200 nm thick) OECT, blue, and 10 µm x 10 µm (50 nm thick) OECT, red, devices showing both drain current (I_D, solid lines), and gate current (I_G; dashed lines) for each device (different shading). Note that the drain current approaches the drain current in the transistor's "off" state.

Supporting Table:

Table S1. Fit results from data in Figure 4b, using the disorder model of main text reference 35.

Material/device	*DOS width (meV)*	*DOS peak position (meV)*	*Percolation fraction (%)*
**p(g2T-TT)** 10x10 μm^2	410 $\pm$ 60	-820 $\pm$ 290	0.1 $\pm$ 0.3
**PEDOT:PSS** 10x10 μm^2	811 $\pm$ 19	-254 $\pm$ 40	1.54 $\pm$ 0.4

Supporting method:

Comparing subthreshold and superthreshold regimes at the same current (I_D):

To achieve the same current for the two regimes of interest we use device geometry engineering: a 100 µm x 10 µm (WxL) transistor, 100 nm thick, shows subthreshold behavior at I_D~1-100 µA, while a 10µmx10µm transistor, 40 nm thick, is in its "superthreshold" region at I_D=100-500 µA ($g_{m,\mathrm{peak}}$=1.6 mS at V_G~-0.6 V). Fig S2a, shows the transfer characteristics of two devices of different dimensions. An operating point of 100µA is selected in order to be within the desired operation regime of both devices (superthreshold for the 10µmx10µm device, and subthreshold for the 100µmx10µm device). Fig S2b shows that while the resulting voltages due to 100 µA operation are not exactly at the peak g_m, or peak g_m efficiency (or alternatively, peak SS), they are acceptably within the desired regime. More precise device engineering could result in a more ideal comparison, however, the present devices can be used as a proof of concept to make the comparisons of the two regimes.

In the above sample set, the 100 µm x 10 µm device, operating at 100 µA (subthreshold) has a transconductance value of 3.5 mS, while the 10 µm x 10 µm device has a transconductance of 0.8 mS at 100 µA. It can be inferred that in this comparison, keeping operating current at 100 µA, the subthreshold device's higher g_m suggests a higher gain can be obtained – in line with the high transconductance efficiency discussed in the main text.

Since voltage output is often required for downstream signal processing, we explore voltage transduction by these devices using the circuit in Fig S2c, inset. A current source shunted with a load resistor (R_L) was used as the load and to set V_{Ds}. The voltage gain in such a configuration can be calculated as $A_V = g_m \times R_L$. The load resistor R_L was chosen to be 20 kΩ, to provide an A_V of 40 to 50 for the device operating at subtheshold. Care was taken to make sure the transistor was in saturation region by only applying a very small sinusoidal input of 1 mV. Similar to AC g_m analysis, the DC operating regions (Q point) of both

the devices, 100 µm × 10 µm and 10 µm × 10 µm p(g2T-TT), was biased at 100 µA by choosing the appropriate V_G voltage. Figure S2c shows the results obtained. The subthreshold region of p(g2T-TT) 100 µm × 10 µm outperformed (5×) the high g_m region of 10 µm x 10 µm device with the same active material in terms of gain. However, the bandwidth was significantly lower, which can be expected due to high gain (constant gain bandwidth product theory).
